# TE-array—a high throughput tool to study transposon transcription

**DOI:** 10.1186/1471-2164-14-869

**Published:** 2013-12-10

**Authors:** Veena P Gnanakkan, Andrew E Jaffe, Lixin Dai, Jie Fu, Sarah J Wheelan, Hyam I Levitsky, Jef D Boeke, Kathleen H Burns

**Affiliations:** 1 The Institute of Genetic Medicine, The Johns Hopkins University School of Medicine, 733 North Broadway, Miller Research Building (MRB) Room 469, Baltimore, MD 21205, USA; 2 The Lieber Institute for Brain Development, The Johns Hopkins Medical Campus, Baltimore, MD, USA; 3 Molecular Biology and Genetics, The Johns Hopkins University School of Medicine, 733 North Broadway, Broadway Research Building, Room 339, Baltimore, MD 21205, USA; 4 Department of Oncology, The Johns Hopkins University School of Medicine, Bunting Blaustein Cancer Research Building, Suite 4M51, 1650 Orleans St, Baltimore, MD 21287, USA; 5 Department of Biostatistics, The Bloomberg School of Public Health, The Johns Hopkins University School of Medicine, Baltimore, MD, USA; 6 Department of Medicine, The Johns Hopkins University School of Medicine, Baltimore, MD, USA; 7 Department of Urology, The Johns Hopkins University School of Medicine, Baltimore, MD, USA; 8 High Throughput Biology Center, The Johns Hopkins University School of Medicine, Baltimore, MD, USA; 9 Department of Pathology, The Johns Hopkins University School of Medicine, Baltimore, MD, USA

**Keywords:** Mobile DNA, Expression microarray, L1 LINE, Endogenous retrovirus, SINE

## Abstract

**Background:**

Although transposable element (TE) derived DNA accounts for more than half of mammalian genomes and initiates a significant proportion of RNA transcripts, high throughput methods are rarely leveraged specifically to detect expression from interspersed repeats.

**Results:**

To characterize the contribution of transposons to mammalian transcriptomes, we developed a custom microarray platform with probes covering known human and mouse transposons in both sense and antisense orientations. We termed this platform the “TE-array” and profiled TE repeat expression in a panel of normal mouse tissues. Validation with nanoString^®^ and RNAseq technologies demonstrated that TE-array is an effective method. Our data show that TE transcription occurs preferentially from the sense strand and is regulated in highly tissue-specific patterns.

**Conclusions:**

Our results are consistent with the hypothesis that transposon RNAs frequently originate within genomic TE units and do not primarily accumulate as a consequence of random ‘read-through’ from gene promoters. Moreover, we find TE expression is highly dependent on the tissue context. This suggests that TE expression may be related to tissue-specific chromatin states or cellular phenotypes. We anticipate that TE-array will provide a scalable method to characterize transposable element RNAs.

## Background

What fraction of mammalian genomes is transcribed has been the subject of intense debate in the scientific literature as different genome-scale platforms have availed themselves to the question [1-6]. In analyzing the transcriptome, non-coding RNAs and products from interspersed genomic repeats have lagged in their characterization as compared to annotated genes. The latter highly repetitive sequences are copies of transposable elements (TEs). Though often dismissed as ‘junk DNA’, cap-selected RNA analyses have suggested that substantial portions of mammalian transcripts originate within these sequences [7], and so they may play significant roles in shaping the functional transcriptome.

**Figure 1:**
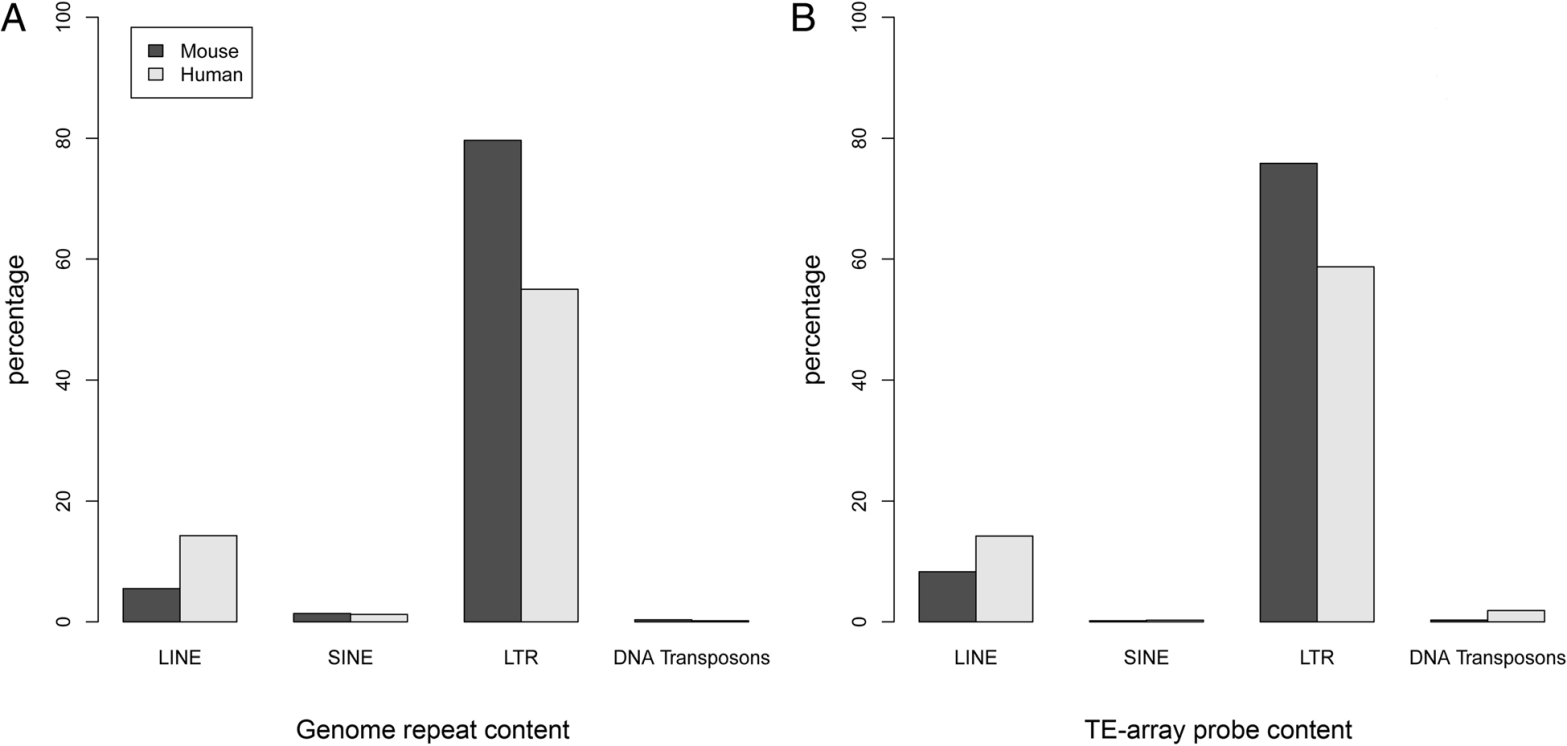
**Species-specific TE families. A)** The genomic contribution of species-specific (rather than ancestral) TE families represented on mouse and human TE-arrays. LTR-based retrotransposons represent the largest class in both species. **B)** The percentage of total TE-array probes for each species-specific array. In mouse, most probes are long terminal repeat (LTR) based TE sequences; these include intracisternal A particle (IAP) and VL30 elements. Long INterspersed Elements (LINE) probes make up the next largest category, although they comprise relatively fewer probes on the murine array as compared to the human array.

While only about 5-10% of our DNA is attributed to coding sequences and putative functional elements, as much as two thirds [8] is recognizable as TE derived. Mammal genomes have been hosts to several kinds of transposons, broadly classified by their method of propagation (reviewed in [9]). Retrotransposons transcribe an RNA intermediate which is then reverse transcribed into DNA inserted at a new location within the genome. Retrotransposons can be subdivided into autonomous and non-autonomous classes based on whether or not they encode proteins that mediate transposition. Major autonomous retrotransposon families include LINEs (Long INterspersed Elements) and ERVs (endogenous retroviruses). Although in modern mammalian genomes most retrotransposon copies reflect ancient insertions rendered incompetent for future transposition, active members exist and promote genetic diversity by generating new insertions [10]. These include LINEs in humans and mice, and intracisternal A-particle (IAP) ERVs in mice. Major non-autonomous families use LINE-encoded proteins for transposition and include SINEs (Short INterspersed Elements) such as *Alu* elements in primates, and B1 and B2 in rodents. DNA transposons propagate through a DNA based ‘cut-and-paste’ mechanism and are extinct in human and mouse. The genomic contribution of transposons varies between organisms. Figure 1A shows the percentage of the ‘mobilome’ owed to each major type of transposon in mouse and human genomes. These proportions reflect homologies of genomic sequence to a manually curated database of repeat consensus sequences, Repbase [11,12].

Hosts have evolved several lines of defense to curb the activity of transposons, including barriers to transcription and means to process transposon-encoded RNAs, though their effectiveness in curbing expression of specific TEs in different contexts are not well described. There are several reasons for researchers to survey TE expression. These include better characterizing pathologic states. For example, massive TE derepression is thought to be cytotoxic, and is associated with male germ cell loss in experimental models and retinal epithelium damage in macular degeneration in humans (reviewed in [13] and [14]). Effects of repeat expression on perhaps a smaller scale may play roles in other diseases - for example, promoting genomic instability in tumors [15-17]. Additionally, derepression of TEs in tumors can generate tumor specific antigens and chimeric transcripts with oncogenic or tumor suppressor activities. Thus, TE-encoded RNAs and proteins may have utility as diagnostic or prognostic markers or as targets for therapy, independent of the ability of the associated TE to promote its own transposition [18-21].

To describe transposon expression in a variety of normal and diseased states, we developed a custom microarray, which we term TE-array. Traditional gene expression microarrays have proved capable of identifying altered TE expression [22-24], but most do not systematically survey numerous types of TEs. We developed platforms tiling all mouse and human TE sequences in both sense and antisense strands. The approach has the advantages of being fairly comprehensive and easily scaled with costs and turn-around times comparing favorably with RNAseq. We envision TE-array as an efficient modality for screening mouse phenotypes and human disease states for changes in patterns of TE expression.

## Results and discussion

### Design of TE-array

To varying degrees, most gene expression microarrays and genomic tiling arrays used for chromatin immunoprecipitation (ChIP-chip) studies ‘mask’ repeat sequences from consideration in probe design or exclude probes without unique matches to the reference genome. Rationales for this include concern that these probes will not behave technically like unique sequence probes (*i.e.,* they will be a source of non-specific high signal) and that they will fail to provide desired information reflective of a specific gene or genomic locus. We are interested in studying transposon-derived sequences, and so we designed an array in which these repetitive probes are the main features.

We designed TE-array probes using annotated consensus sequences of *Mus musculus* or *Homo sapiens* specific repeats from Repbase [11,12]. Probes were obtained using a custom PERL script to collect 60 bp sequences across the length of each repeat consensus. Overlapping probes were collected with offsets of 14-15 bp increments over short repeats, and every 30-45 bp for long repeats. For the sense strand arrays, the probes are the same strand (*i.e.,* the identical sequence) as the consensus sequence found in Repbase [11,12]. We also created counterpart arrays for antisense strand detection by using the reverse complement of each probe sequence. We did not duplicate highly similar probe sequences, culling probes from the list with fewer than 3 bp mismatches to an already accepted probe using BOWTIE and un-gapped BLAST alignments. The contribution of different TE families to total genomic repeat content for the two species is shown (Figure 1A). The representation of each family with respect to probes on TE-array mirrors these proportions (Figure 1B). For all of the experiments described, we prepared labeled RNA samples for hybridization to TE-array in accordance with standard methods for gene expression arrays. A schematic of both TE-array design and RNA sample labeling is shown in Additional file 1: Figure S1.

### Array reproducibility

To test the reproducibility of TE sequence probe behavior, we carried out a series of technical and biological replicates. Technical replicates involved multiple hybridizations of single RNA preparations. We used RNA from breast cancer cell lines derived from Balbc mice overexpressing rat Her2/Neu for these studies, N3D and N1G. Briefly, adherent cells were expanded, total RNA extracted, cDNA labeled in parallel Cy3-dCTP and Cy5-dCTP reactions, and sense strand TE-arrays hybridized for 17 hours. Variability in dye behaviors was normalized using the widely applied local regression (LOESS) method [25-27]. The M value, defined as the log_2_ ratio of the normalized Cy3 and Cy5 fluorescent intensities, was evaluated at each probe. As expected, the M values from technical replicate comparisons scatter at M value = 0 (median M value = 0.00; s.d. = 0.21 for N1G). Additional file 2: Figures S2A and B show the pairwise correlation plots within each replicate type and cell line.

Two types of biological replicates were performed; the first compared different RNA preparations from independent cultures of the same cell line. In these studies, we labeled one RNA sample using Cy3 and its corresponding sample using Cy5; and hybridized these together (Additional file 2: Figure S2C and D; M value s.d. = 0.33 and 0.47, respectively). The second approach used RNA isolated from different dissections of the same mouse organ compared to RNA from a pool of different mouse organs; results of these studies are described further below. Additional file 3: Table S1 describes the number of replicates used in each phase of this study.

### Detection of expressed genes and transfected transposons

We included gene-specific probes on the microarray as positive controls for reverse transcription and hybridization conditions. As expected, these probes showed previously described patterns of gene expression. Tissue specific expression was most striking for several testis genes (Additional file 4: Figure S3).

As a positive control for TE expression detection, we transfected human HeLa cells with a plasmid to exogenously express Long INterspersed Element 1 (LINE-1 or L1). L1 sequences account for about 17% of the human genome, and L1 is of special interest as a subset are active today in humans and mice [28-31]. There are an estimated 500,000 copies of L1 in the human genome, although most are small fragments. Endogenous L1 transcripts and splice products have been reported as a series of faint bands in Northern blots of HeLa cells using a 5′UTR L1 probe [32].

**Figure 2:**
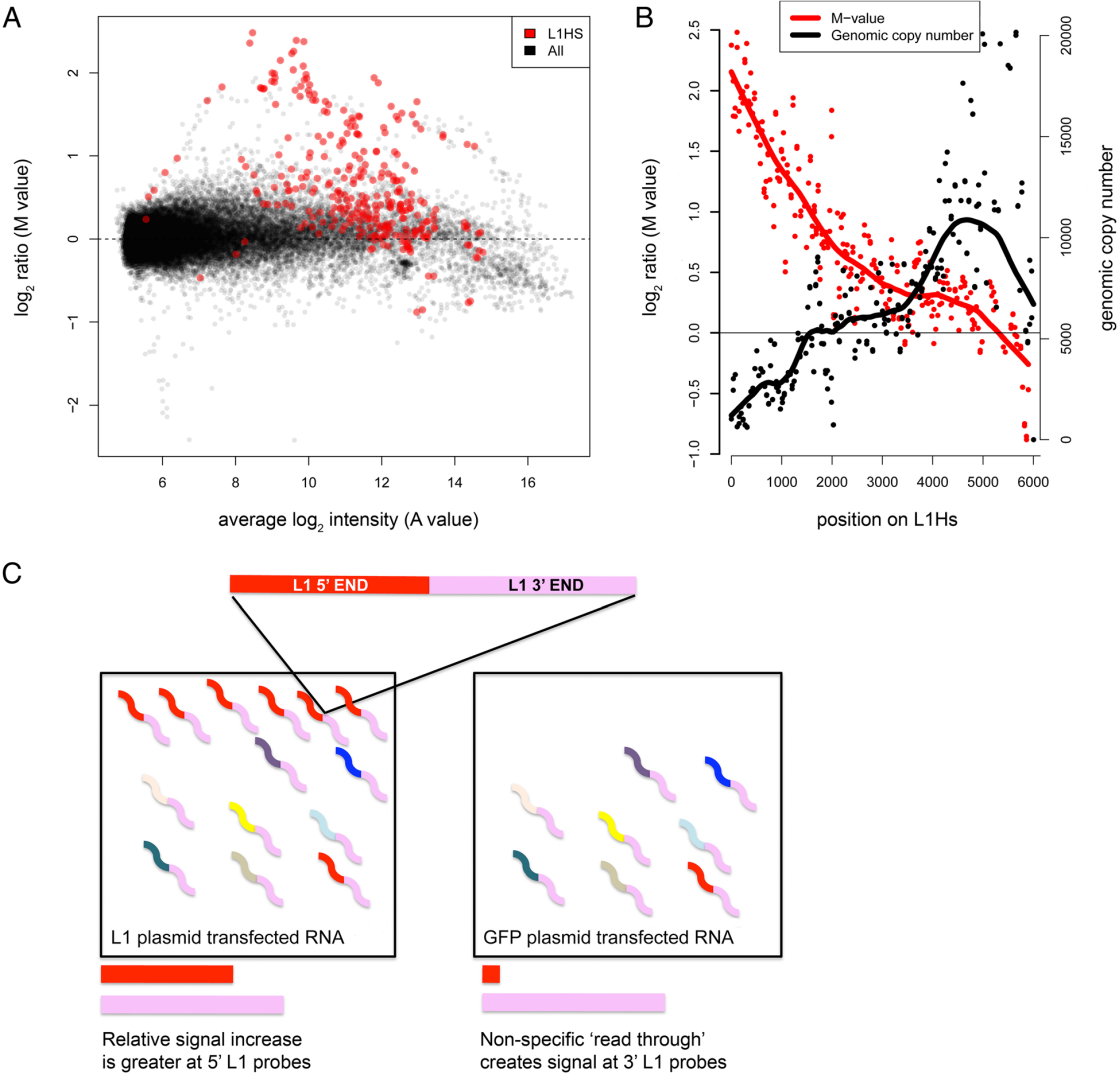
**Exogenous L1 transfection experiment. A)** Shown is an MA plot of TE array signals from L1 transfected HeLa cells as compared to untransfected cells. The y-axis shows the M value (log_2_ ratio) for each probe; the x-axis shows the A value, which is the average of the log_2_ intensities from the two channels. Black dots represent all TE probes. Red dots represent L1HS probes (*Homo sapiens*-specific L1). The L1 probes detect elevated transcript abundance from cells with the L1 plasmid. **B)** L1 expression map. M values are shown relative to probe positions as they occur along the length of a full-length, 6 kb, LINE-1. The highest signal is detected using probes at the 5′ end of the element, whereas the 3′ end has relatively similar signal transfected and control cells (red line). Overlaid is a graph depicting genomic copy number of L1Hs sequences relative to their position in the L1Hs consensus (black line). Copy number increases along the length of the L1 consensus sequence until the 3′ end; here, divergences of the 3′UTR sequence that distinguish young L1 subfamilies from older L1 sequences occur, and the smoothed curve is drawn downward. **C)** This graphic shows how the M value is greatest in probes mapping to the 5′ end of the L1 (colored red). Since most L1 elements in the genome are 5′ truncated, 3′ end mapping L1 fragments (colored pink) can be incorporated into transcripts of other RNA species (multicolored lines). This is seen in both GFP and L1 transfected cells, whereas, full length L1 RNA copies (5′ is red and 3′ end is pink) are abundant in L1 transfected cells. The lengths of the horizontal red and pink bars below represent the abundance of 5′ and 3′ L1 RNAs respectively.

To test the ability of TE-array to detect ectopically expressed L1, L1Hs (human-specific L1) and (negative control) GFP- expressing plasmids were transiently transfected into HeLa cells. Polyadenylated RNA was extracted from both cultures, and an RT-PCR was performed to verify L1 expression from the plasmid. This assay generates an amplicon only from the ectopically expressed L1 transcript, which includes non-L1 5′UTR sequence before sharing the remainder of its sequence (including all protein coding sequences) in common with endogenous L1. Each RNA sample was then fluorescently labeled with either Cy3 or Cy5, and these were co-hybridized to the TE-array. Results are shown in Figure 2A-C. As expected, we detected L1 as the “top hit” in L1Hs plasmid transfected cells (*i.e.,* the TE type with the greatest probe M values) as compared to cells with the control plasmid. Figure 2A shows an MA plot of all probes on the array. This graph plots a measure of the change in expression, the M value (a log_2_ ratio of the dye signals), against the average of the log_2_ of the fluorescence intensities, the A value. Probes with very high signals in the control changed relatively little (*i.e*., M values trended lower at high A values), while significant shifts in M values were seen for other L1Hs probes (red). While most non-L1 TEs and repetitive sequences did not change in this study (black dots, Figure 2A), L1 transfection was also associated with downregulation of the spliceosomal U1 snRNA. This may be related to decreased L1-snRNA chimeric RNAs [33], though we have not tested this.

To better understand why a subset of L1Hs probes showed changes in expression in this experiment, we plotted the M value of each probe verses its copy number in genomic DNA (Figure 2B). Full length (6 kb) L1 genomic copies are infrequent, and most are 5′ truncated. This observation has been ascribed to interruptions in the reverse transcription (RT) and integration of new L1 sequences; RT initiates at the 3′ L1 end [34]. Thus, the 3′ L1 end is present in several hundred thousand genomic copies, whereas the 5′ end occurs a few thousand times in our genome (probes mapping to regions 750 bp and upstream of an L1 human specific (L1Hs) element average at 6774 copies in the human genome) (Figure 2B, black line). We saw the highest M value increments with L1 expression selectively at 5′ L1 probes (Figure 2B, red line). The effect was pronounced; probes mapping to the 3′ end were not significantly different in the cells transfected with L1 as compared to control GFP-transfected cells. Since many of the latter probes showed high fluorescence intensity in both transfected and untransfected samples, we propose that these probes reflect chimeric transcripts or non-specific ‘read through’ RNAs that incorporate the 3′ end of L1. A schematic of these presumptive products (present in L1-transfected and control cells) and the relatively greater increase in 5′ L1 RNA sequences with transfection is shown (Figure 2C).

### TE-array ‘top hits’ show concordance with nanoString^®^ and RNAseq analyses

#### *(i) nanoString^®^ concordance*

NanoString is an RNA quantification method based on the ability of RNA molecules to bridge complementary, sequence-specific ‘capture’ and ‘reporter’ probes [35].

**Figure 3:**
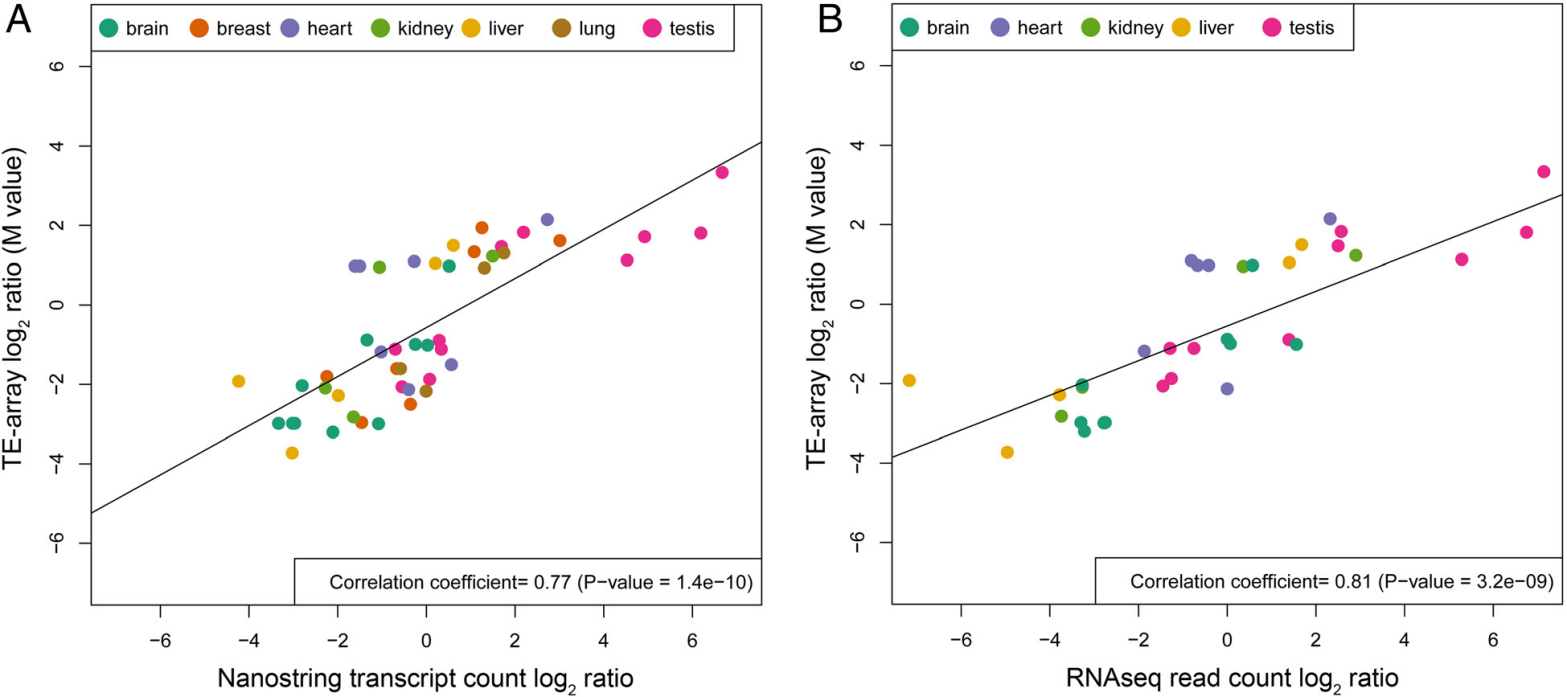
**Cross platform TE-array validation. A)** NanoString probes were designed for 30 candidate TE regions shown to be differentially expressed in at least one tissue by TE-Array. Discrete transcript counts were obtained for individual tissues and for the somatic pool. Log_2_ ratios of transcript counts were compared to TE-array M values. **B)** RNAseq reads were aligned to the 30 candidate TE regions. Log_2_ ratios of tissue read counts were generated in comparison to an *in silico* pool; these are plotted on the X-axis, and M values from TE-array are plotted on the Y-axis.

To test concordance between this method and TE-array, we selected 30 TE regions found to be significantly differentially expressed in TE-array experiments in at least one tissue as compared to the somatic tissue pool. Abundance of these regions in polyA RNA from individual tissues (lung, liver, testis, brain, kidney, heart, and breast) was then measured using nanoString probes. Polyadenylated RNA from the same pool of tissues used in the TE-array studies was used for comparisons. Log_2_ ratios of TE sequence counts in the individual tissue RNAs verses in pooled RNA were calculated and compared to TE-array log_2_ ratio (M value). The correlation plot comparing these two methods is shown in Figure 3A; Pearson’s correlation coefficient was 0.77 (p = 1.4 × 10^-10^).

#### *(ii) RNAseq concordance*

The same 30 TE regions were also evaluated using publicly available RNAseq data [36]. TE aligning read counts were acquired using sequencing of polyadenylated RNA from individual tissues as well as an *in silico* generated RNA pool. The log_2_ ratios of read counts were compared to TE-array M values. The average Pearson’s correlation coefficients between all tissue comparisons is 0.81; data are shown in Figure 3B.

### Strand bias in TE RNA levels

Antisense specific TE arrays have special utility to reveal transcription initiated from internal TE antisense promoters as well as instances of repeat-containing transcripts generated by ‘read-through’ transcription with TE ‘exonization’, which can happen in either direction along the length of a repeat unit. In some cases, internal TE antisense promoters (ASP) are known and may be expected to reproducibly generate signal at corresponding probes. An example is an ASP in the 5′ end of human L1s [37]. Exonization events, in which an intronic or gene proximal TE is expressed by ‘read through’ and retention of TE sequence in a cellular transcript would map in a highly locus-dependent manner, with intervals of expression bounded by any functional splice sites along the length of the TE.

**Figure 4:**
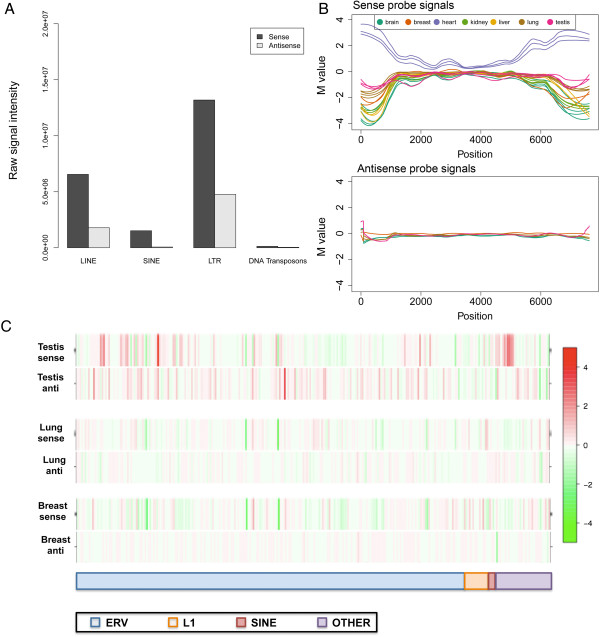
**Strand bias of TE transcripts. A)** Bar plot showing the sense strand bias of TE expression. Total raw signal intensities of TE probes are plotted for sense and antisense orientations. **B)** An ERV2 member, IAPEY4_I is segementally expressed in the sense strand in heart. Corresponding antisense probes show no differential expression across tissues. The Y-axis of this plot shows the log_2_ ratio value and the X-axis shows positions of the probe with reference to the element sequence. **C)** Heatmap of sense versus antisense experiments of testis, lung, and breast. The X-axis represents the mouse TE families ordered by type (blue for ERVs, orange for L1s, red for SINEs and purple for all other families). The mean M value (log_2_ ratio) of all probes is assigned to each family is shown, the values of which range between -4 and 4.

To compare global sense verses antisense profiles of TE expression, we ran testis, lung, breast, and brain samples on both antisense and sense detecting microarrays. Antisense array probes showed overall lesser intensities and far fewer examples of differential TE expression than did the sense arrays. The relative gap in intensities is illustrated by Figure 4A. Probes corresponding to L1 and LTR retrotransposons in antisense showed average raw signal intensities of approximately 30-40% the values measured for sense probes of the same elements. For short interspersed repeat families (SINEs), this effect was more pronounced; average antisense signal intensities were only 5% of sense signals. The top 1000 probes as ranked by signal intensity detecting L1, LTR, and SINE RNA in sense had 5.2 fold, 3.4 fold, and 27.7 fold, respectively the average intensity of the top 1000 antisense probes. This suggests that the bulk of TE expression occurs in a sense strand specific manner relative to the TE. Alignments of ultra-high-depth, stranded RNAseq data to the mouse Repbase consensus sequences reflect a more modest overall (+) strand bias in our hands, most evident in species-specific repeat types as opposed to ancestral repeats in the mouse genome [56.2% (+) strand; data not shown].

Similarly, most instances of differential (tissue-specific) TE expression were seen in the sense orientation. An example expression plot shows this (Figure 4B). Here, distinct portions of the IAPEY4_I endogenous retrovirus consensus sequence can be seen to be expressed in heart. This is unique to the sense strand (top panel, slate blue line) and not evident when the antisense strand is considered (lower panel). The M value (log_2_ ratio) is used as the metric for differential expression in this plot.

M values for more TEs are represented by red/green color intensities for sense and antisense detecting arrays (Figure 4C). Of the tissues studied, testis showed relatively more expression of antisense TE transcripts as compared to somatic tissues, an effect we consider further in the discussion.

### TE transcripts show tissue-specific expression patterns

To investigate how expression patterns vary with tissue type, we profiled sense TE expression in a panel of normal mouse tissues, including lung, heart, liver, kidney, brain, breast, and testis.

**Figure 5:**
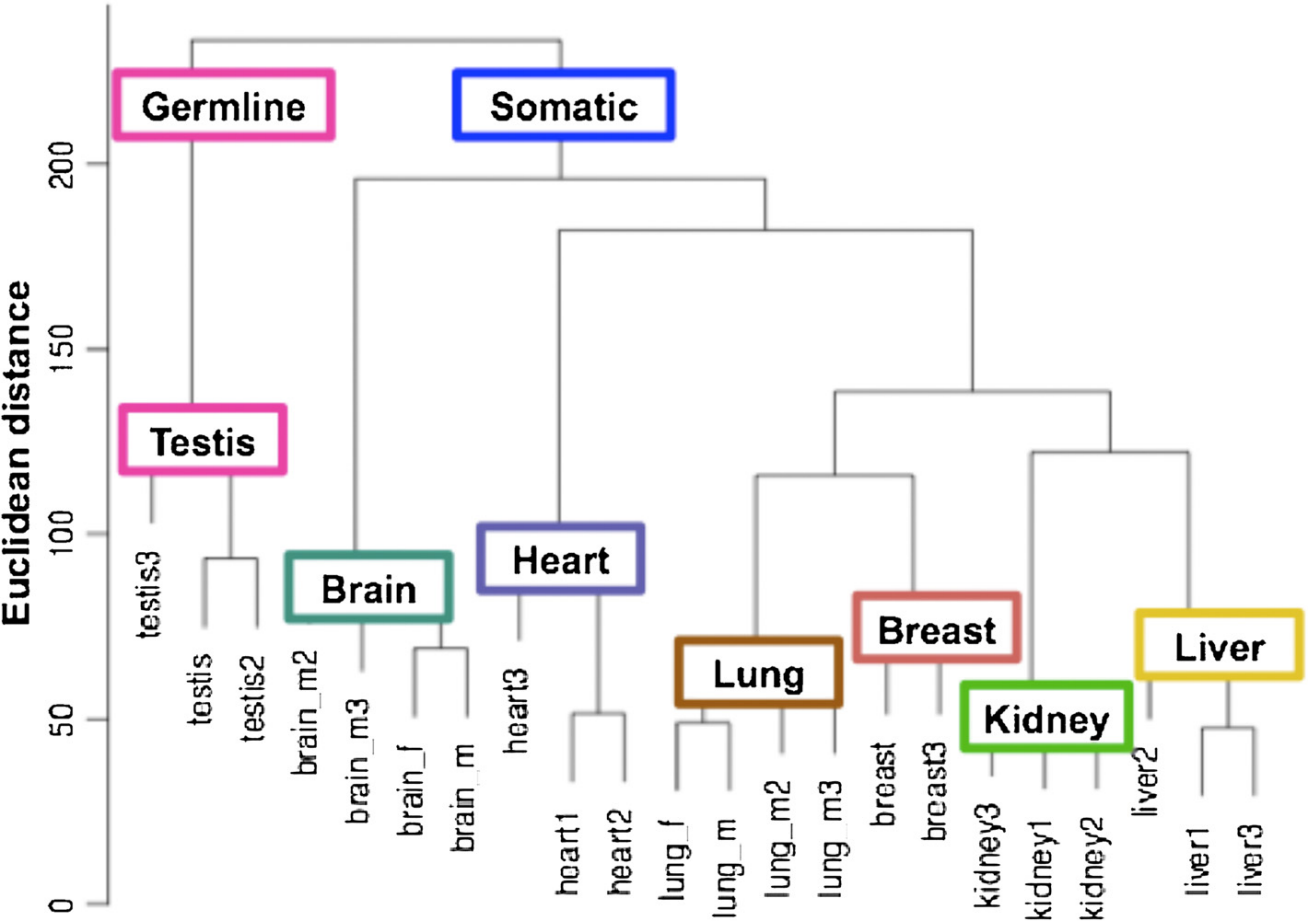
**Tissue-specific TE expression.** The dendrogram shows Euclidean (pairwise) distances between germline (testis) and somatic (brain, heart, lung, breast, kidney and liver) tissues. The most distinct TE expression profile is seen in testis while somatic tissues cluster together. Among the latter, brain and heart are somewhat separated from the other somatic tissues.

TE expression profiles of individual samples were compared using a Euclidean distance function. The resulting dendrogram showed tissue types could be delineated based on their expression of repeat-containing RNA (Figure 5). The profile is most distinct in comparing germ line (testis) tissue to the various somatic tissues. To generate a different visual, the same Euclidean distance matrix was used for a multidimensional scaling (MDS) plot (Additional file 5: Figure S4).

**Figure 6:**
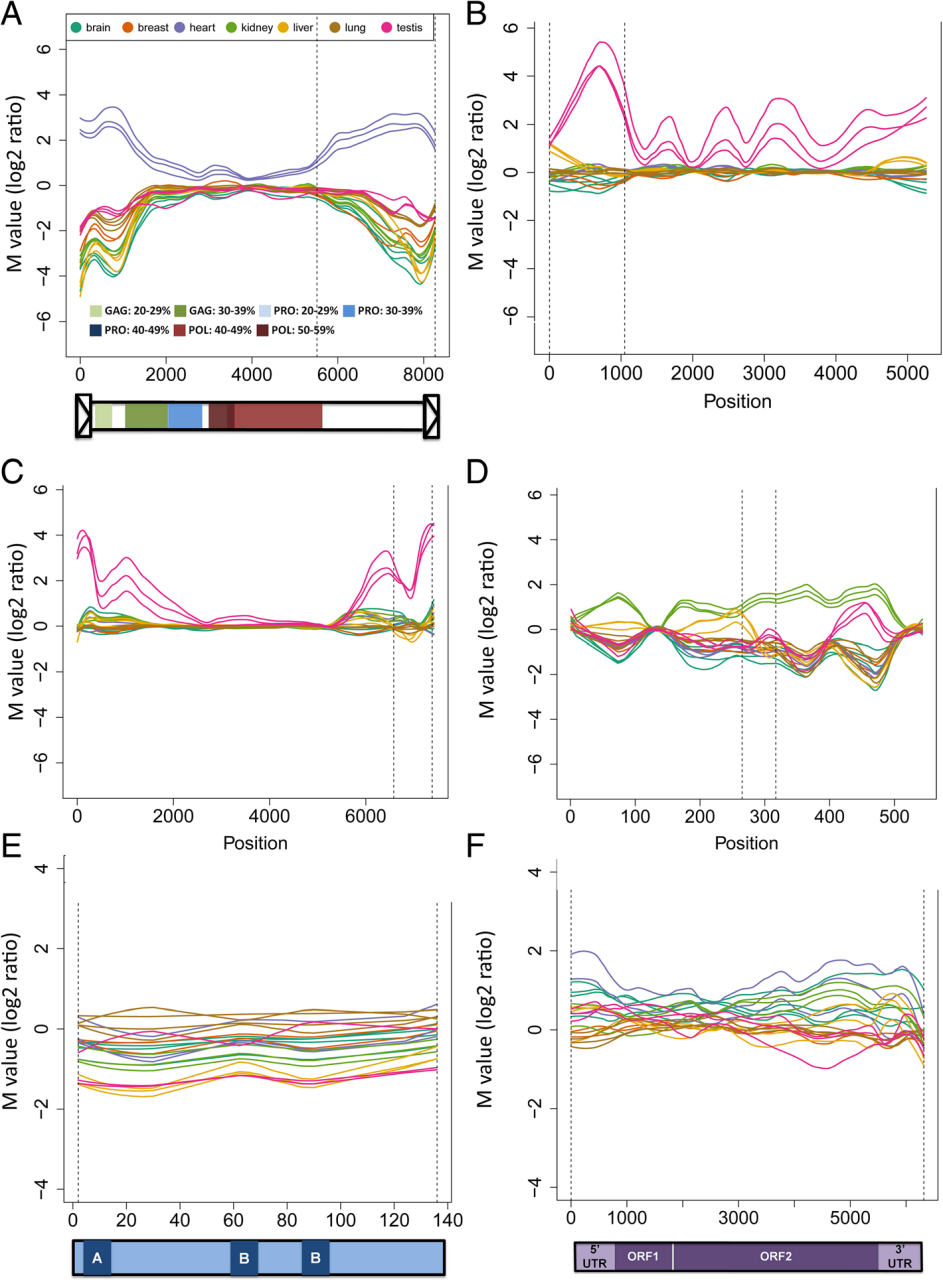
**Expression maps of differentially expressed TEs.** Differentially expressed portions of TEs are shown with respect to their location in the element consensus sequence; the y-axis shows the log_2_ ratio of signals between the indicated tissue type and the pool of tissues, and the x-axis shows the corresponding position in the TE. **A)** An ERV2 member, IAPEY4_I is variably abundant in heart. IAPEY4 LTR sequences (which are separate Repbase entries) were added to proviral sequences for a complete depiction of the element. Annotation of the functional domains gag, pol and env were made based on Blastx alignments to Mtv1; darker hues of a given color indicate a higher percent identity of similarity to the exogenous virus. The annotation of (b) and (c) are similar; (d) is a long terminal repeat (LTR) of an endogenous retrovirus with no ORFs. **B)** ERVB4_1B, another endogenous retrovirus, shows testis specific segmental expression. **C)** IAPLTR3 also an ERV2 shows segmental transcript abundance in testis as well. **D)** RLTR1D2 is an ERV1 long terminal repeat which shows differential transcript abundance in kidney. **E)** B1 SINE is a non-autonomous and non- LTR TE which does not show highly tissue specific abundance. **F)** LINE-1 or L1, an autonomous, non-LTR TE without a highly tissue specific abundance.

Deviations of M values (log_2_ ratios) away from zero of contiguous TE probes were used to identify highly tissue specific TE expression. Figure 6 shows expression maps of several highly differentially expressed mouse endogenous retroviruses as well as a representative murine L1 and SINE. These graphs show M values (log_2_ ratios) on the y-axis plotted against the TE consensus sequence length.

Finally, we tested whether TE sequences may be expressed in a tissue-specific manner because of co-regulation with surrounding tissue-specific genes. Disproportionate numbers of these TE sequences embedded in differentially expressed gene loci would be an indication of such co-regulation. To assess this, we recovered genomic positions corresponding to differentially expressed TE segments and annotated each for occurrence within a gene transcript unit. For a given tissue type, this provided a list of gene loci containing sequences of differentially expressed TEs. We saw no overrepresentation of differentially expressed genes as compared to randomly selected genes in these lists (data not shown).

## Conclusions

Measuring expression from highly repetitive genomic sequences is complex. Part of this is owed to inherent features of genomic repeats. Many TE families have populated mammalian genomes over evolutionary time, and members of each type have shown fragmentation and degenerating homology with aging. A second layer of complexity stems from variant transcript structures. Interspersed repeat sequences can be transcribed actively as well as passively as a part of a cellular pre-mRNA or other forms of cellular RNA, and can then be processed by RNA splicing machinery with a variety of outcomes [32]. Thus, though methods like Northern blots [38], RNase protection assays [39], RT-PCRs [40], and RACE PCRs [41] all suggest interesting patterns of repeat expression in human tissues and cells, they thus far have focused on a very minor subset of elements (often, those competent for transposition). They cannot be practicably scaled to capture the complexity of the ‘mobilome’ in a variety of tissue types.

We thus approached the task of designing a TE expression microarray platform. For both human and mouse repeats, we have designed an 88,000 probe, two array set that is available for distribution to laboratories through the manufacturer. Probes have been designed against all annotated transposons - approximately 300 mouse and 500 human TE consensus sequences - in both sense and antisense orientations. Coverage is dense and deep (~10X on average), with probes sequences designed in overlapping intervals along the length of each TE consensus sequence. We address numerous technical concerns in this publication, showing that these probes do not generate arbitrary irreproducible signals and that there is agreement between TE-array and other state-of-the-art RNA detection modalities, including nanoString and RNA-seq.

Using TE-Array to profile transposon expression in a variety of normal mouse tissues, we make two encompassing observations. The first is that transposon expression is detectable most frequently from probes for sense strand transcripts. This expression is most often segmental or involving short series of contiguous probes along the length of longer repeat sequences. We interpret the strand bias as evidence that sequences intrinsic to the interspersed repeats themselves are directing much of this transcription, consistent with the observation that many transcripts originate in this portion of the genome [7]. In contrast, exclusively ‘read through’ transcription of intronic TEs as directed by gene promoters would be expected to give comparable signals in sense and antisense for most element types where there is random orientation of each TE within its gene locus.

Our second major observation is that there are highly distinct patterns of retroelement expression in crosswise comparisons of different mouse tissues. Specific intervals of a broad complement of TE families appear differentially expressed in any given tissue. These patterns of sense strand repeat expression appear pervasive; they do not only typify testis, where specific TE repression pathways have been described or brain, where L1 LINE expression has been studied (reviewed in [10]). Interestingly, although testicular samples adhere to the sense strand bias we describe above, examples of intense signals from antisense probes are more prevalent in this tissue. It is possible these signals reflect primary antisense transcripts from TEs or piRNA clusters which initiate piRNA biogenesis [42]. piRNAs in turn are critical for imposing TE silencing during gametogenesis. Spermatogenic tubule dissection experiments may demonstrate stage specificity for these antisense species and provide insights into their regulation.

Our work complements and extends observations made with the first generation of microarrays to detect interspersed repeats, RepArray, designed by Horard et al. [43]. Using 236 oligonucleotides, their design designates a single oligonucleotide target and its reverse complement for each type of transposable element. Each probe appears in duplicate on the array. The key advantage of our platform design relates to probe quantity; TE-array comprises in contrast 88,000 probes. A ten kilobase retrovirus is given a single sense-oriented and antisense-oriented probe pair on RepArray chosen to maximize specificity for that family. The same element is represented by more than 650 overlapping, sense and antisense oriented probes on TE-array. Given how TE expression can be seen over discrete intervals within a larger consensus sequence, this density of coverage appears vital to capture. Excitingly, RepArray has proven useful not only as an expression array, but also in transcription factor chromatin immunoprecipitation (ChIP) and methylated DNA immunoprecipitation (MeDIP) experiments [44]. Although these applications may be highly sensitive to the lack of probe coverage on this platform, the experiments are a demonstration of how microarrays can give insights into TE regulation at multiple molecular levels.

Our discovery of regulated, differential TE expression in tissues – from sequences incapable of transposition and in somatic cells where there is no consequence for TE propagation in the population – might be viewed as an unexpected result from the perspective of transposon biology. Whether these are ‘exapted’ elements with functional roles in tissues is unknown, though their discovery begins to make this question experimentally tractable. Moreover, outside of normal cell differentiation and function, profiling TE expression in cancers, infertilities, and degenerative conditions where repeat derepression has been described may suggest functional roles in disease or provide new markers of a disease state.

In summary, we show that a wide diversity of transposon sequences can be incorporated in expression microarray designs to provide more comprehensive profiles of TE expression. First applications of the technology suggest that control of TE expression is determined by repeat encoded sequence features and regulated in a highly tissue-specific manner.

## Methods

### RNA extraction from cell lines and tissues

Normal tissue experiments used 12 week old C57BL/6 mice. Animals were sacrificed. The tissues were dissected and flash frozen on dry ice. Tissue subsections were homogenized in Trizol (Invitrogen, Carlsbad, CA) and total RNA was extracted and ethanol precipitated (Ambion, Carlsbad, CA). RNA was quantified using Nanodrop (Thermo Fisher Scientific, Wilmington, DE) and its integrity measured using Bioanalyzer 2100 (Agilent, Santa Clara, CA) according to the manufacturer’s directions. Normal somatic tissue RNA pools of 6 μg were made by combining 600 ng of RNA from each of the following tissues: lung (2 male animals, 300 ng each and 1 female animal, 600 ng), brain (3 males, 200 ng each and 3 females 200 ng each), liver (1 male, 600 ng and 1 female, 600 ng), kidney (1 male, 600 ng and 1 female, 600 ng) and heart (1 male, 600 ng and 1 female, 600 ng).

Cell line experiments used human HeLa (subclone HA) and mouse N1G, N2C and N3D, all three of which were derived from primary breast cancer overexpressing Rat Her2/Neu in Balbc mice [45].

### L1HS transfection and qRT-PCR

HeLa-HA cells were maintained in Dulbecco’s Modified Eagle Medium (D-MEM) supplemented with 10% fetal bovine serum and 100units/ml penicillin/streptomycin. HeLa were seeded in 6-well plates (2×10^5^ cells/well) the day before transfection. pLD107 (pCEP puro -eGFP) was made by cloning eGFP gene into pCEP puro plasmid after the CMV promoter (between *Nhe*I and *BamH*I sites). pLD190 (pCEP puro L1.3-GFPAI) was made by replacing L1RP sequence (*Not*I/*BstZ*17I fragment) within pLD223 [46] with L1.3 fragment from JM101 (*Not* I/*BstZ*17I) [47]. The next day each well was transfected with 1 μg of either pLD107 or pLD190 plasmid using Fugene 6 (Roche Applied Science, Indianapolis, IN) according to the manufacturer’s protocol. The day after transfection, cells were trypsinized and transferred to a 6 cm plate with 4 mL DMEM medium containing puromycin (2.5 μg/ml). After 3 days of puromycin selection, total RNA was extracted using RNeasy mini kit (Qiagen, Valencia, CA) according to the manufacturer’s protocol. For qRT-PCR, 1 μg RNA was used for cDNA synthesis using Superscript^®^ III reverse transcription kit (Invitrogen, Carlsbad, CA) in a 20 μL reaction. Real time PCR was performed using a 1 μl cDNA sample as template in a 20 μL reaction on a Step One Plus instrument (Applied Biosystems, Carlsbad, CA). Primers used for real time PCR were: Beta actin gene: JB12931, JB12932; L1: JB14148, JB14149, JB14150. The 2-DDCT method was used for normalization to the β-actin mRNA level.

### Microarray probe design

Mouse and human specific TE consensus sequences were obtained from Repbase [11,12] and 100 genes were selected as controls, including oncogenes, tumor suppressors, and housekeeping genes. As probe design algorithms employed by eArray (Agilent technologies, Santa Clara CA) do not lend themselves to high resolution coverage of interspersed repeats, we obtained 60 bp subsequences across TE consensus and control gene sequences without this program. TE families with consensus sequence less than 1 kb long were tiled every 14-15 bp and those greater than 1 kb long were tiled with overlapping probes sequentially offset in 30-45 bp increments without consideration of base composition. For control genes, the probe mapping to the first 60 bp of each exon was selected. The reverse complement of the exact same probe sequences were taken for the antisense detecting arrays. Probe sequences with ambiguous bases were discarded as were probes with >57 bp identity to a probe already present on the array. BOWTIE mapping [48] and ungapped BLAST alignments were used to determine identical probes. Probe sequences were submitted to Agilent technologies (Santa Clara, CA) to print on a 4 × 44 K microarray platform. Four distinct designs were submitted (Table 1). These designs (probe identifiers and sequences) are available for public download or for ordering from the manufacturer; probe identifiers and their corresponding genomic copy numbers are available in Additional file 6: Table S2.

**Table 1:** Agilent Microarray Design Identifiers for TE-arrays

**Species**	**Strand detected**	**Agilent Microarray Design Identifier (AMADID)**
Mouse	Sense	025451
Mouse	Antisense	025450
Human	Sense	025411
Human	Antisense	025412

### RNA labeling and microarray hybridization

Poly-A RNA was reverse transcribed to double stranded cDNA using MMLV RT. T7 promoter was ligated to the 3′ end corresponding to poly-A. T7 was used to generate single stranded, labeled cRNA. RNA was labeled using Quick amp kit (Agilent) with Cy-dye labeled Cytosine. Arrays were hybridized following the manufacturer’s protocol for 17 h. Longer hybridization times up to 65 h were attempted in order to verify saturation at the recommended 17 h. Arrays were washed and scanned on an Agilent 2 μm scanner at 70% PMT (photomultiplier tubes) for both green and red channels.

### Microarray data analysis

Fluorescence signals were preprocessed and normalized using the *limma* software package from R/Bioconductor [27]. Specifically, probe intensities from the two-channel arrays were log_2_ transformed and LOESS normalized. Raw intensity plots suggested high hybridization efficiency (not shown). Subsequent analyses were performed on the normalized log_2_ fold changes of each probe/transposable element relative to a control channel (M value). This can be considered a measure of relative expression abundance. For example, M = 1 for a given tissue and repeat family corresponds with that family having twice as much expression in the experimental sample (*i.e.,* RNA of a specific tissue or cells with L1 expression) as compared to a reference sample (*i.e.,* RNA of a multi-tissue pool or cells after a control transfection).

We performed “bumphunting” algorithm, adapted from microarray-based DNA methylation data [49,50], to identify transposon families showing expression. First, M values were smoothed within each family using LOESS, a smoothing function robust to outliers [25], with a smoothing window of approximately 100 base pairs - this within-family smoothing was performed for each family. An F-statistic was computed at each probe from the smoothed M values to identify differential expression by tissue type (statistical model: *M*_*ijk*_ = *α*_*i*_ + ∑ _*k*_*β*_*ik*_*X*_*jk*_ + *ϵ*_*ijk*_ for probe *i*, biological replicate *j*, and tissue type *k* where X is an indicator for tissue type, compared to the intercept only model: *M*_*ij*_ = *α*_*i*_ + *ϵ*_*ij*_). Then we performed thresholding to identify contiguous probes within a given transposable element family that were differentially expressed by tissue type. We therefore identify differentially expression regions from differentially expressed probes – each region was summarized by the sum of its F-statistics within the region (an “area” statistic) and ranked by this area.

### Cross platform comparisons

We compared the “top hits” called by TE-Array using nanoString (nanoString technologies, Seattle, WA) and publicly available RNA sequencing (RNAseq) data.

#### *(i) nanoString comparison*

A non-redundant subset of 30 “top hit” regions (>200 bp long) was selected along with positive control regions from 3 housekeeping genes (*Oaz1*, *Rpl27* and *Rps13*). These sequences were submitted to nanoString for probe design as per the company’s protocols. The probes and 200 ng of RNA from 11 tissues and the pool of somatic tissue RNA were run on the nCounter. Discrete transcript counts from individual tissues and the somatic tissue pool were obtained. Log_2_ ratios comparing individual tissues to the somatic pool were determined and compared to the corresponding TE-array M values.

#### *(ii) RNAseq comparison*

Previously described [36] RNAseq data were chosen to match the genetic background and approximate age of mice used in our experiments. We used RNAseq data from testis and male and female brain, lung, liver, kidney and heart. Tissue specific reads were aligned to extracted segments of TE consensus sequences corresponding to the “top hit” interval using Bowtie [48] allowing for at most 3 mismatches. For comparison, we generated an *in silico* pool of somatic tissue (male and female brain, lung, liver, kidney and heart) RNA reads by averaging aligned read counts of the included tissues, and calculated log_2_ ratios of each individual tissue alignable read count to this average. This was compared to the corresponding M value from TE-Array and the Pearson’s coefficient of correlation was calculated.

### Data access

The data discussed in this publication have been deposited in NCBI’s Gene Expression Omnibus and are accessible through GEO Series accession number GSE52412 (http://www.ncbi.nlm.nih.gov/geo/query/acc.cgi?acc=GSE52412).

## Abbreviation

TE: Transposable element.

## Competing interests

The authors declare that they have no competing interests.

## Authors’ contributions

JDB, KHB, HIL, SJW, and VPG designed the study. VPG, JDV and SJW designed the microarray. VPG performed tissue culture, RNA extractions, microarray procedures in the Burns lab and conducted downstream bioinformatics analysis with AJ. LD constructed plasmids and carried out the L1HS transfections in the Boeke lab. JF harvested mouse tissues from the Levitsky animal colony. VPG, KHB, AJ, LD and JDB wrote the manuscript. All authors edited and approved the manuscript.

## Supplementary Material

Additional file 1: Figure S1 (Left) Array design. Mouse or human specific TE consensus sequences were tiled with 60 bp probes. (1) TE families with consensus sequence less than 1 kb long were tiled every 14-15 bp with overlapping probes; (2) those greater than 1 kb long were tiled with probes sequentially offset in 30- 45 bp increments. (Right) Poly-A RNA (strand 1) was reverse transcribed to double stranded cDNA using MMLV RT (strands 2 and 3). T7 promoter was ligated to the 3′ end corresponding to poly-A, and T7 was used to generate single stranded, labeled cRNA. RNA was labeled using Cy-dye labeled Cytosine (strand 4). Click here for file

Additional file 2: Figure S2 **A)** Technical replicates of the mouse N3D cell line. Four replicate TE-array hybridizations were performed with aliquots of RNA extracted from one cell culture. Plotted are pairwise correlations showing the behavior of all probes for each replicate type. **B)** Three technical replicates of RNA from the N1G cell line. **C)** Biological replicates. Four independent N3D cell cultures were expanded for RNA extraction and TE-array hybridization. **D)** Six biological replicates of N1G cells. Click here for file

Additional file 3: Table S1 A: Replicates, B: Tissues. Click here for file

Additional file 4: Figure S3 Tissue specific gene expression. As a control for reverse transcription and hybridization conditions, 100 genes were chosen and an array probe placed in each gene exon. Shown are M value (log_2_ ratio) plots for two testis specific genes, *Brdt* (bromodomain testis-specific protein) **(A)** and *Theg* (testicular haploid expressed gene) **(B)**. Click here for file

Additional file 5: Figure S4 Intra- and Inter-tissue clustering of TE-array and gene expression (GE) data. Multidimensional scaling applied to Euclidean distance was used to categorize tissues using **A)** TE-array and **B)** traditional gene expression microarray data. Click here for file

Additional file 6: Table S2 The Excel worksheet has 4 tabs corresponding to different versions of TE-array. Abbreviations denote the species (hs, *Homo sapiens*; mm, *Mus musculus*) and strand (as, antisense strand; ss sense strand) of the version. Each list provides the probe ID and its genomic copy number. Corresponding sequences can be downloaded from the manufacturer’s site. Click here for file
