# Supplementary figures and images for: TE-array—a high throughput tool to study transposon transcription
Source: BMC Genomics. 2013 Dec 10;14:869. doi: 10.1186/1471-2164-14-869 (PMC3878892; doi:10.1186/1471-2164-14-869)

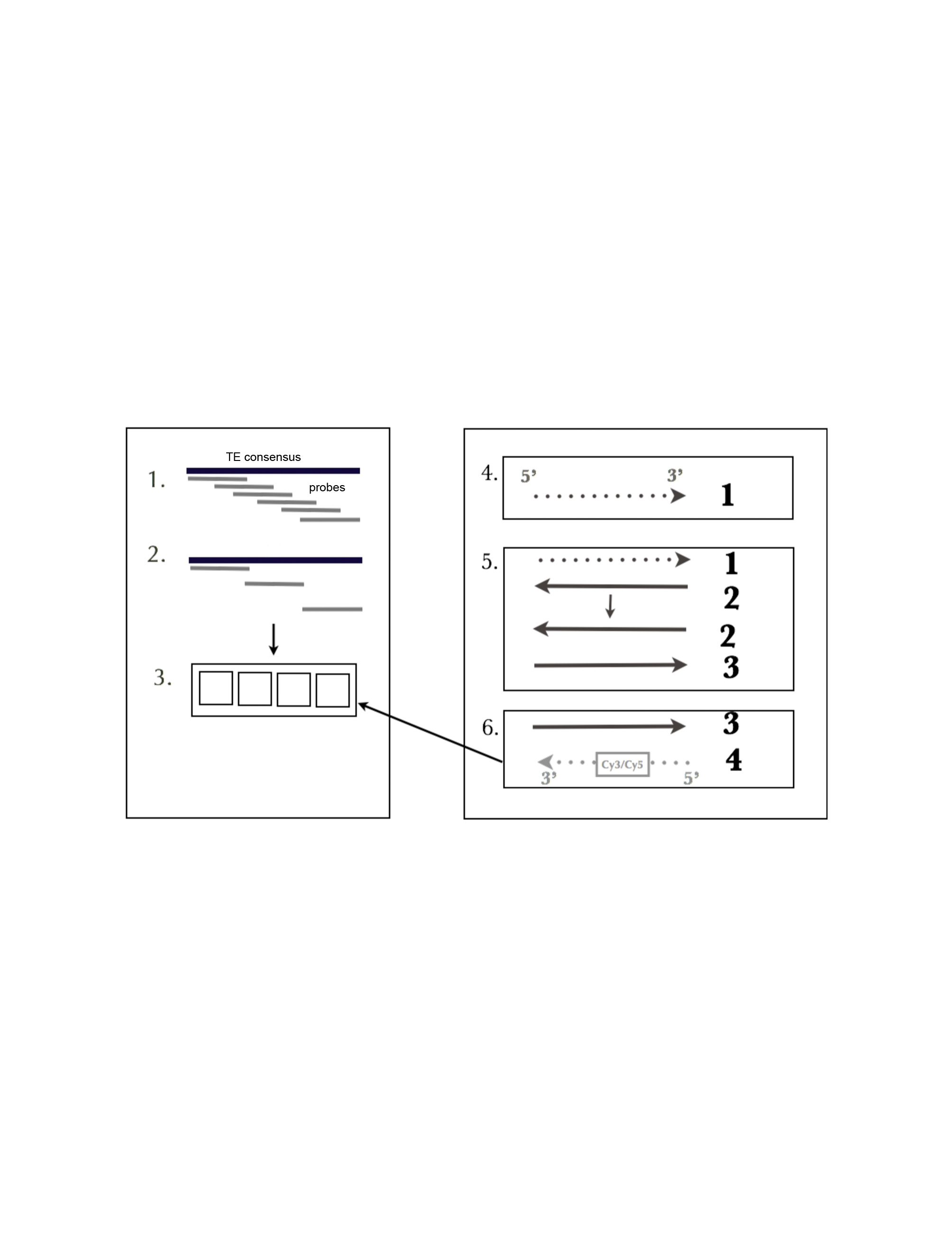

Supplement: Additional file 1: Figure S1 — (Left) Array design. Mouse or human specific TE consensus sequences were tiled with 60 bp probes. (1) TE families with consensus sequence less than 1 kb long were tiled every 14-15 bp with overlapping probes; (2) those greater than 1 kb long were tiled with probes sequentially offset in 30- 45 bp increments. (Right) Poly-A RNA (strand 1) was reverse transcribed to double stranded cDNA using MMLV RT (strands 2 and 3). T7 promoter was ligated to the 3′ end corresponding to poly-A, and T7 was used to generate single stranded, labeled cRNA. RNA was labeled using Cy-dye labeled Cytosine (strand 4). [file 1471-2164-14-869-S1.jpeg]

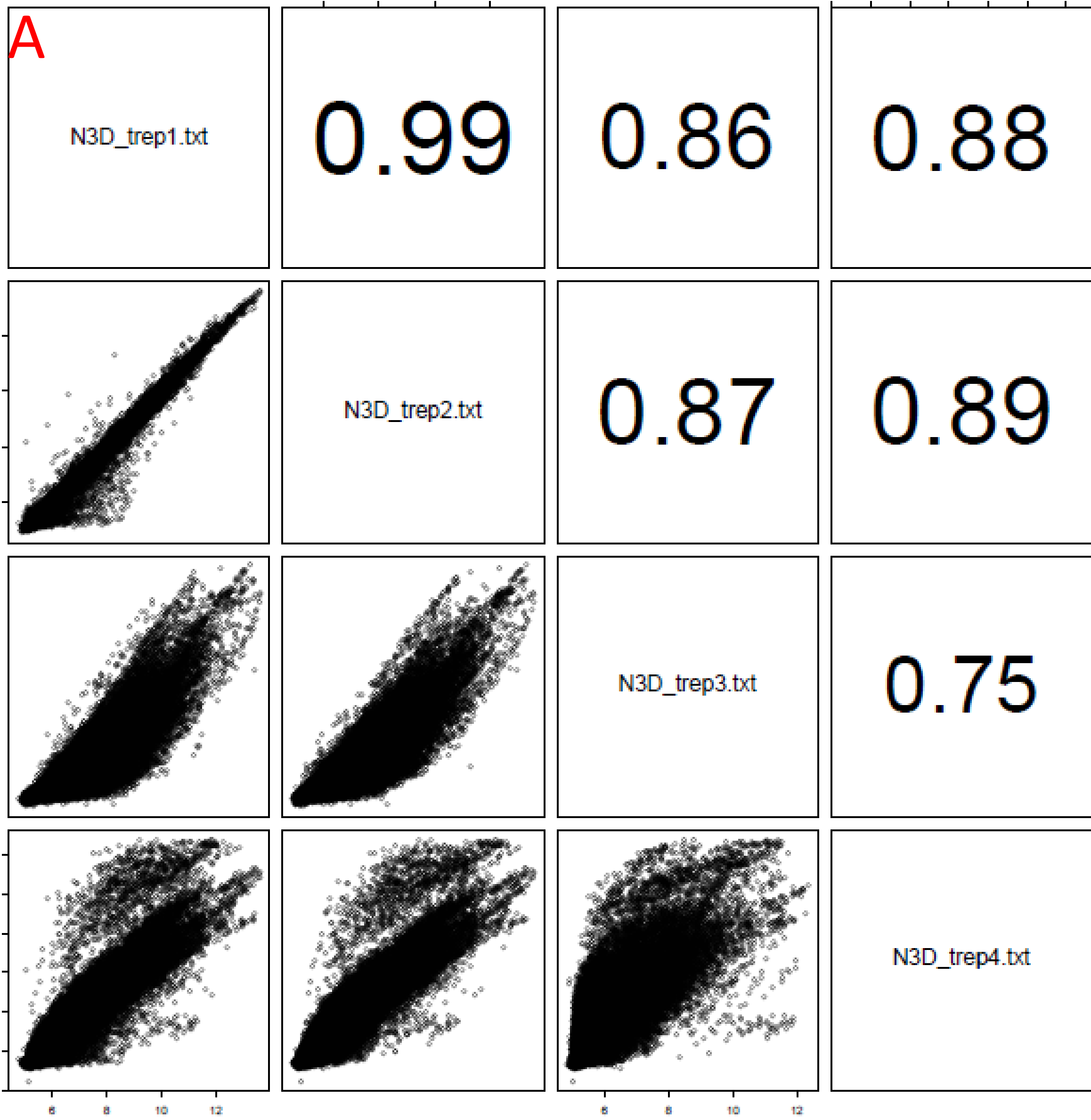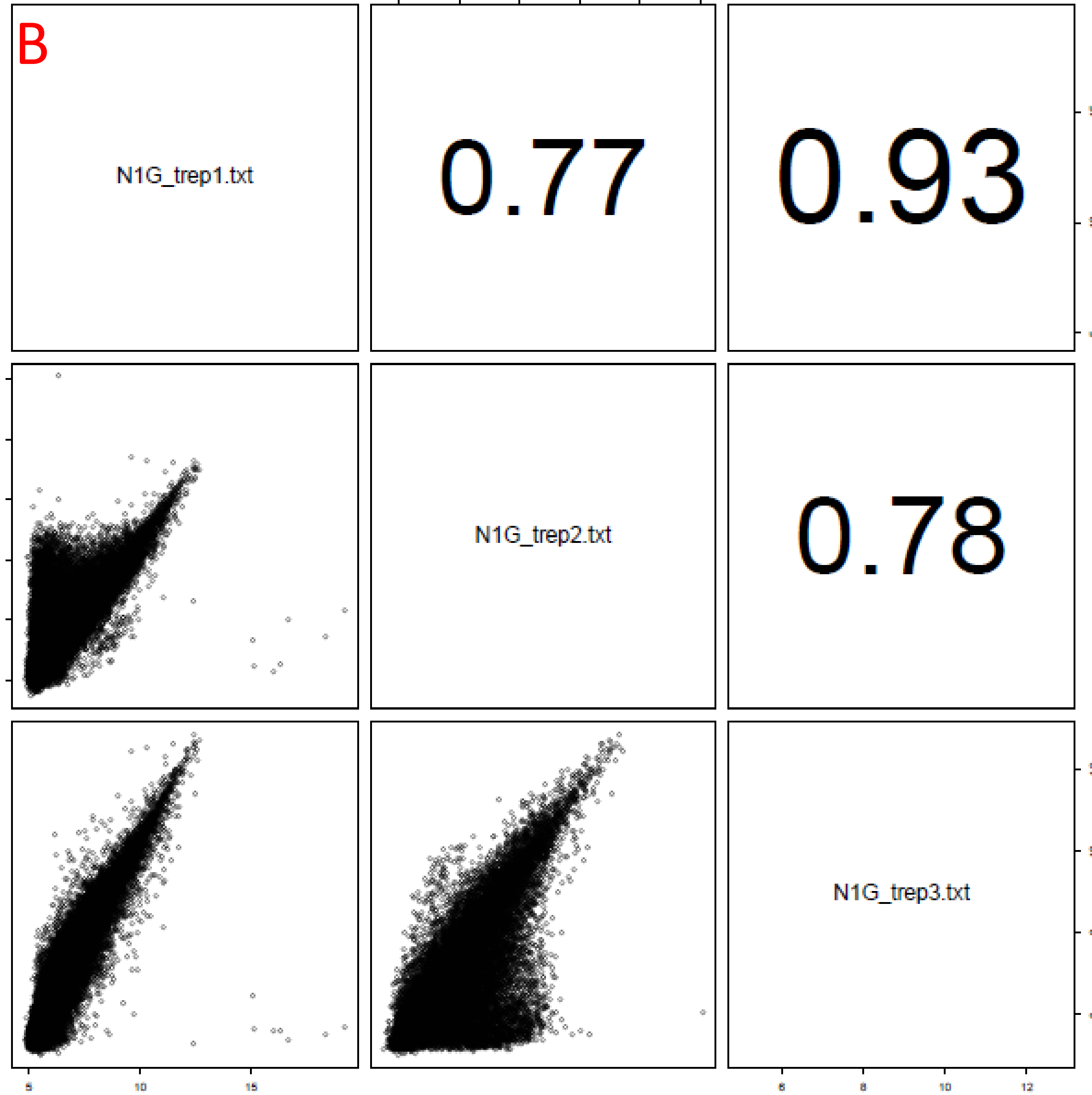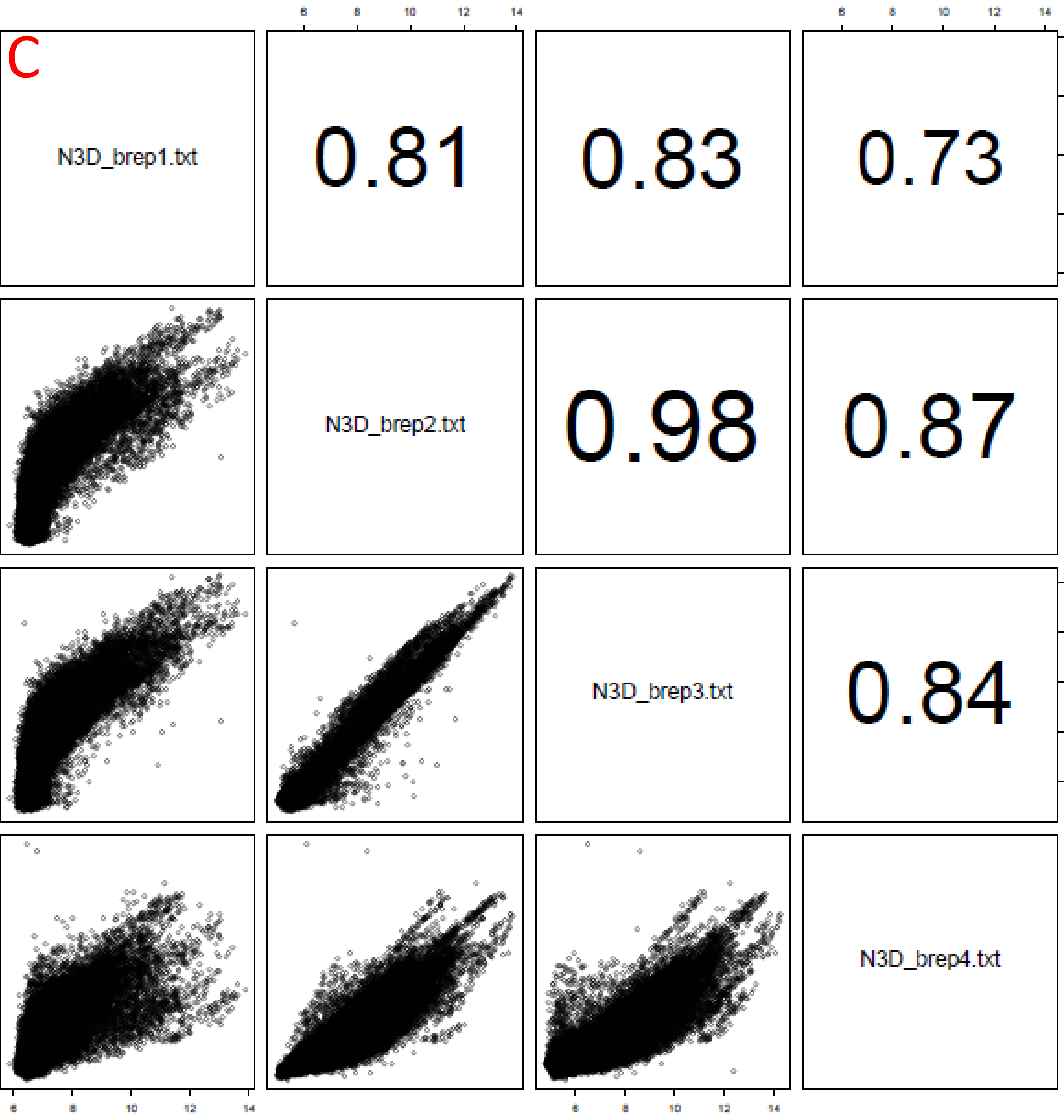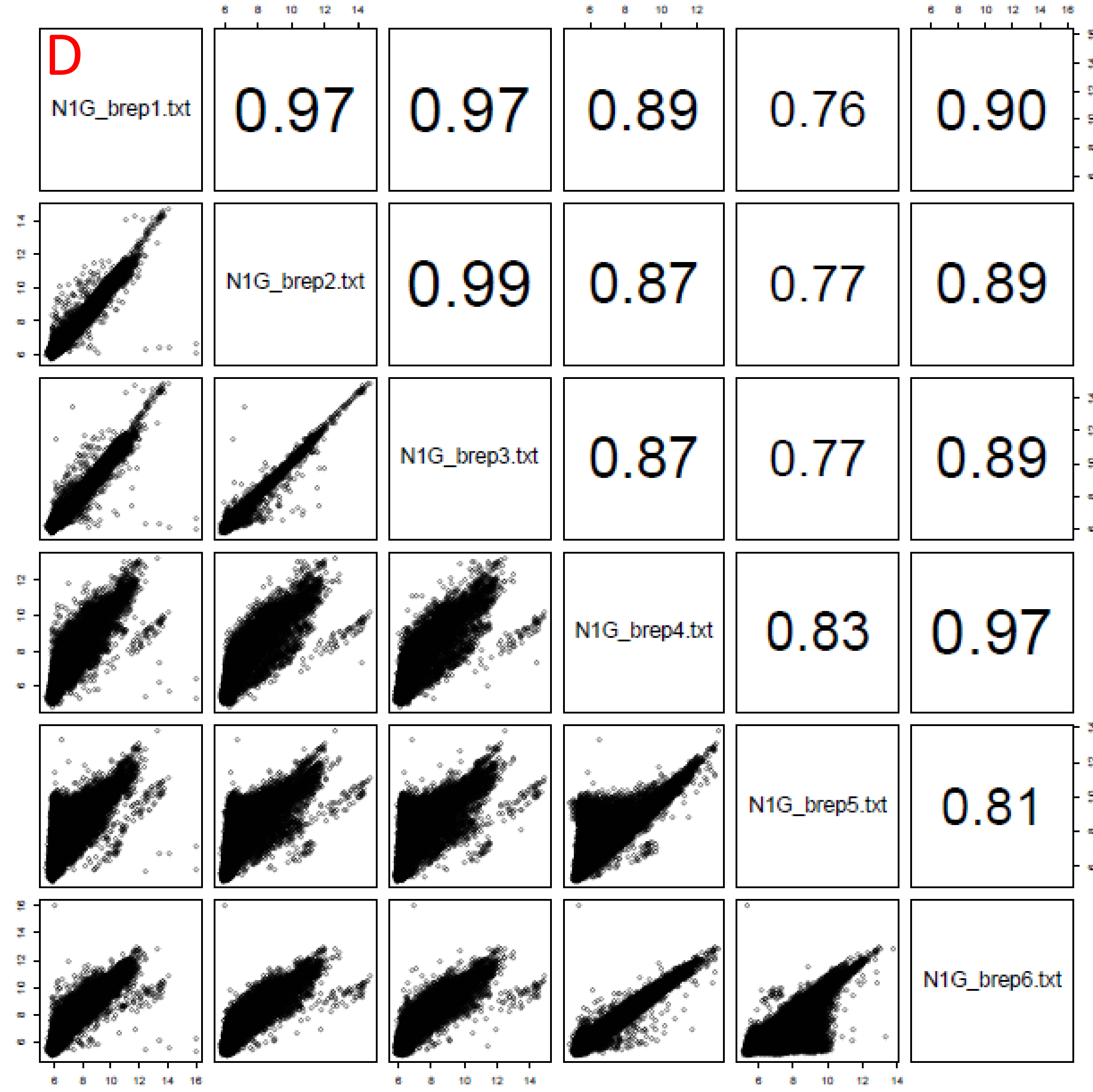

Supplement: Additional file 2: Figure S2 — A) Technical replicates of the mouse N3D cell line. Four replicate TE-array hybridizations were performed with aliquots of RNA extracted from one cell culture. Plotted are pairwise correlations showing the behavior of all probes for each replicate type. B) Three technical replicates of RNA from the N1G cell line. C) Biological replicates. Four independent N3D cell cultures were expanded for RNA extraction and TE-array hybridization. D) Six biological replicates of N1G cells. [file 1471-2164-14-869-S2.pdf]

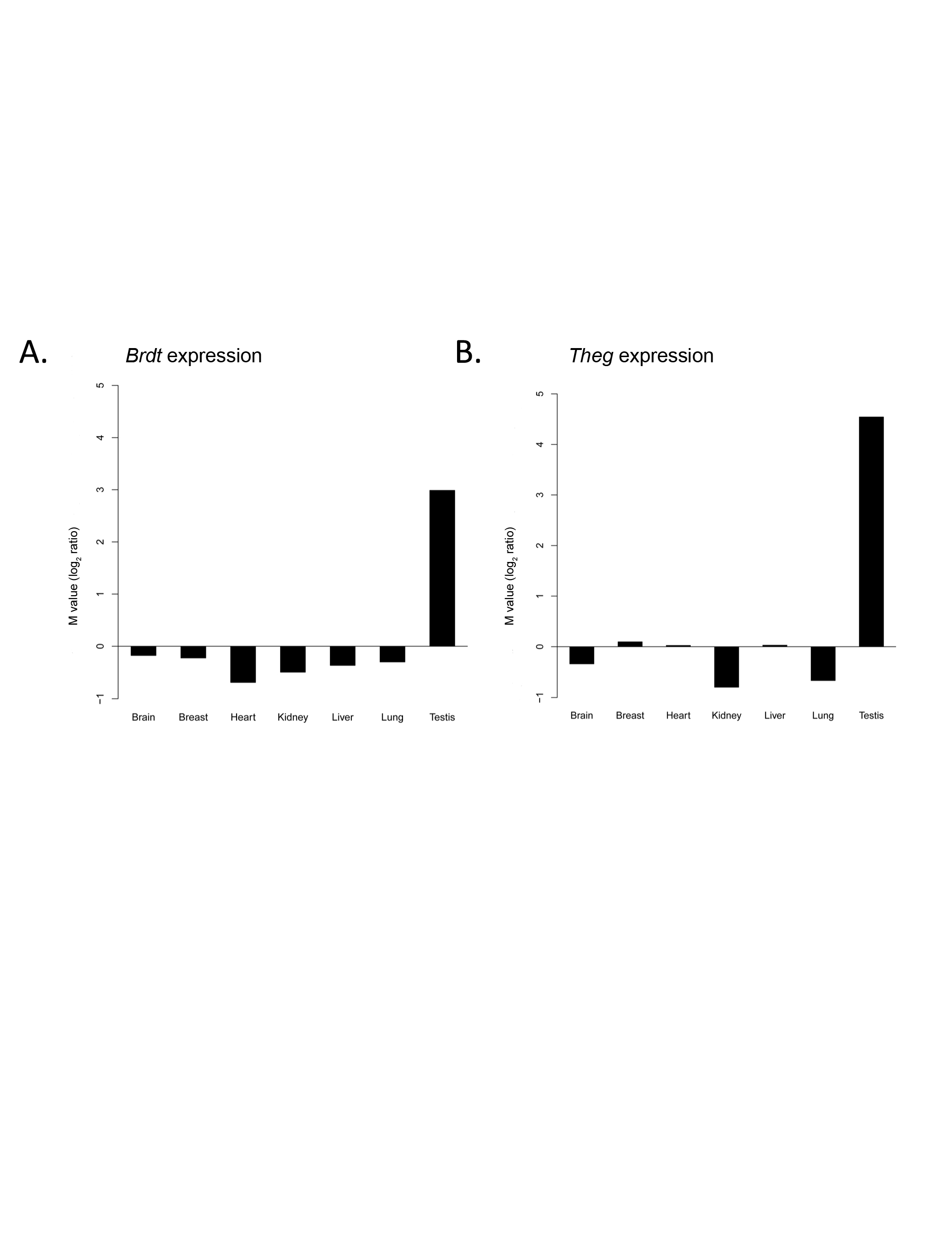

Supplement: Additional file 4: Figure S3 — Tissue specific gene expression. As a control for reverse transcription and hybridization conditions, 100 genes were chosen and an array probe placed in each gene exon. Shown are M value (log2 ratio) plots for two testis specific genes, Brdt (bromodomain testis-specific protein) (A) and Theg (testicular haploid expressed gene) (B). [file 1471-2164-14-869-S4.jpeg]

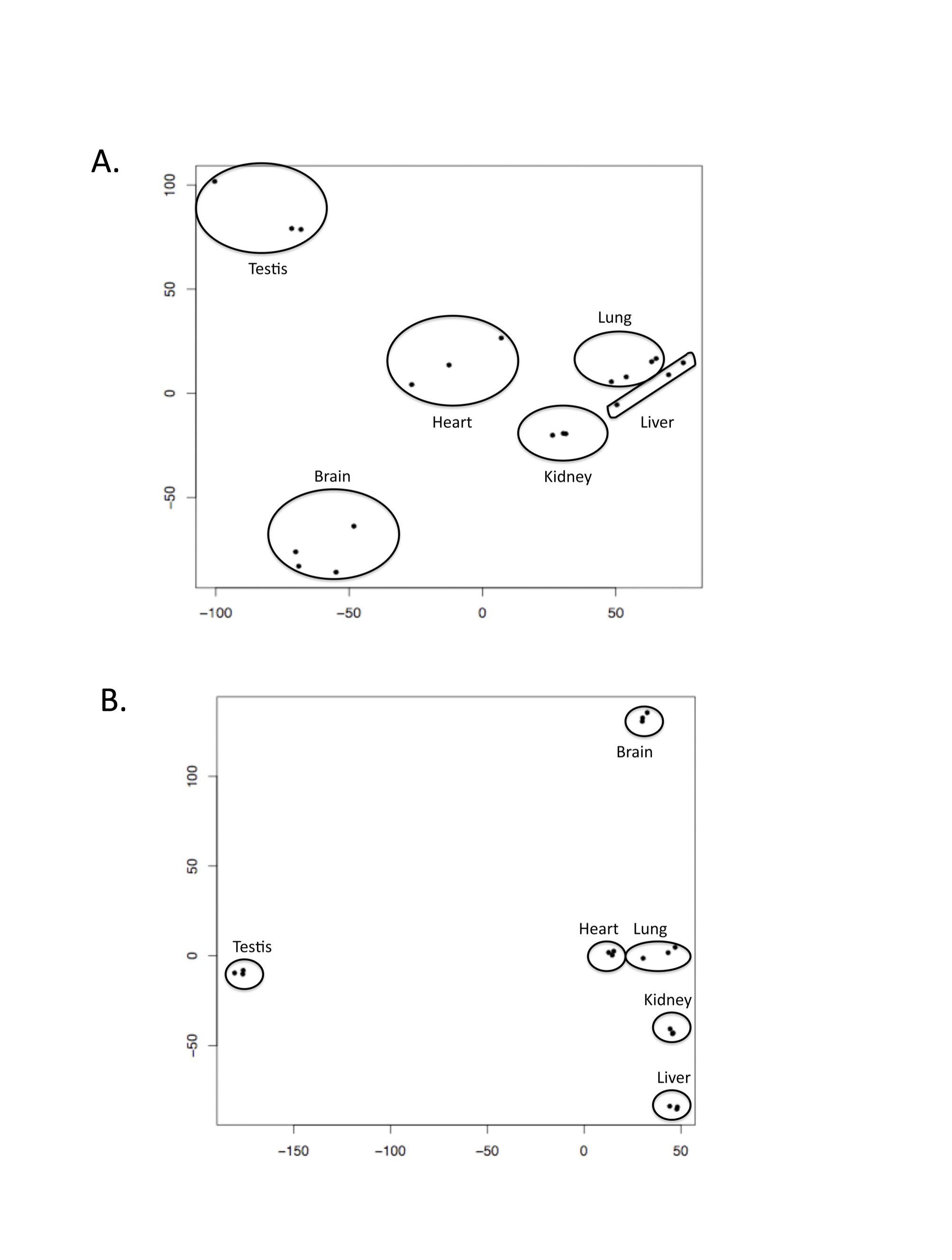

Supplement: Additional file 5: Figure S4 — Intra- and Inter-tissue clustering of TE-array and gene expression (GE) data. Multidimensional scaling applied to Euclidean distance was used to categorize tissues using A) TE-array and B) traditional gene expression microarray data. [file 1471-2164-14-869-S5.jpeg]
